# “Pharmakognosie – Phytopharmazie”

**DOI:** 10.3797/scipharm.br-10-01

**Published:** 2010-06-30

**Authors:** Astrid Obmann

**Affiliations:** Department of Pharmacognosy, University of Vienna, Austria

The 9^th^ new edition of “Pharmakognosie – Phytopharmazie” provides a detailed overview of plant derived natural products with respect to phytochemistry and phytopharmacology. Basic knowledge on phytochemistry, biosynthesis of active plant metabolites and screening methods, as well as special pharmaceutical aspects, e. g. preparation of herbal medicinal products, quality control, and quality assurance of plant extracts and herbal medicinal products are presented. Additionally, allergies or other side effects related to the application of herbal remedies, and the role of secondary plant metabolites as food supplements are discussed. A short chapter is dedicated to TCM and explains not only basic principles of diagnosis, preparation of TCM-drugs and their therapeutic use, but also aspects concerning quality and safety.

However, the main part of the book deals with the description of herbal drugs used in western medicine and their active ingredients such as terpenoids, alkaloids, phenolics, carbohydrates, lipids etc. with respect to chemical analysis and pharmacological activities. For each chapter a detailed list of references is available online (pdf-download). Within 1451 pages (including 731 figures) the reader is given a comprehensive overview of plant secondary metabolites with regard to pharmaceutical aspects. Even for the non German speaking reader this book represents an excellent reference source containing also critical remarks, and therefore can be highly recommended to everybody interested in pharmacognosy!

